# The genome and transcriptome analysis of snake gourd provide insights into its evolution and fruit development and ripening

**DOI:** 10.1038/s41438-020-00423-9

**Published:** 2020-12-01

**Authors:** Lili Ma, Qing Wang, Jianlou Mu, Anzhen Fu, Changlong Wen, Xiaoyan Zhao, Lipu Gao, Jian Li, Kai Shi, Yunxiang Wang, Xuewen Zhang, Xuechuan Zhang, Zhangjun Fei, Donald Grierson, Jinhua Zuo

**Affiliations:** 1 Key Laboratory of Vegetable Postharvest Processing, Ministry of Agriculture, Beijing Key Laboratory of Fruits and Vegetable Storage and Processing, Key Laboratory of Biology and Genetic Improvement of Horticultural Crops (North China) of Ministry of Agriculture, Key Laboratory of Urban Agriculture (North) of Ministry of Agriculture, The Collaborative Innovation Center of Cucurbit Crops, Beijing Vegetable Research Center, Beijing Academy of Agriculture and Forestry Sciences, Beijing, 100097 China; 2 College of Food Science and Technology, Hebei Agricultural University, Baoding, 071001 China; 3 Beijing Advanced Innovation Center for Food Nutrition and Human Health, Beijing Technology and Business University, Beijing, 100048 China; 4 Department of Horticulture, Zhejiang University, Hangzhou, 310058 China; 5 Biomarker Technologies Corporation, Beijing, 101300 China; 6 Boyce Thompson Institute for Plant Research, Cornell University, Ithaca, NY 14853 USA; 7 U.S. Department of Agriculture-Agricultural Research Service, Robert W. Holley Center for Agriculture and Health, Ithaca, NY 14853 USA; 8 School of Biosciences, University of Nottingham, Sutton Bonington Campus, Loughborough, Leicestershire LE12 5RD UK

**Keywords:** Conservation genomics, Next-generation sequencing

## Abstract

Snake gourd (*Trichosanthes anguina* L.), which belongs to the Cucurbitaceae family, is a popular ornamental and food crop species with medicinal value and is grown in many parts of the world. Although progress has been made in its genetic improvement, the organization, composition, and evolution of the snake gourd genome remain largely unknown. Here, we report a high-quality genome assembly for snake gourd, comprising 202 contigs, with a total size of 919.8 Mb and an N50 size of 20.1 Mb. These findings indicate that snake gourd has one of the largest genomes of Cucurbitaceae species sequenced to date. The snake gourd genome assembly harbors 22,874 protein-coding genes and 80.0% of the genome consists of repetitive sequences. Phylogenetic analysis reveals that snake gourd is closely related to sponge gourd but diverged from their common ancestor ~33–47 million years ago. The genome sequence reported here serves as a valuable resource for snake gourd genetic research and comparative genomic studies in Cucurbitaceae and other plant species. In addition, fruit transcriptome analysis reveals the candidate genes related to quality traits during snake gourd fruit development and provides a basis for future research on snake gourd fruit development and ripening at the transcript level.

## Introduction

As the second largest vegetable family, the Cucurbitaceae family comprises one of the most genetically diverse groups of plants. The members of this family are prevalent in tropical regions^1,2^ and many species are now cultivated worldwide as food crops. Snake gourd (*Trichosanthes anguina* L.; 2*n* = 2× = 22) is a diploid annual woody climber (liana) of the genus *Trichosanthes* in the family Cucurbitaceae^2^. This species is commonly called viper gourd, snake tomato, or long tomato, and has several synonyms (e.g., *Trichosanthes cucumerina* L.). Snake gourd originated in India or the Indo-Malayan region in tropical Asia^3,4^ and is widely distributed in Asian countries^5–7^. Its green, tender stems, leaves, and fruits are consumed as edible vegetables^2,6^, which have high nutritional value, because they are rich in vitamins, essential minerals, dietary fiber, and other nutrients, and are a wholesome, healthy addition to diets^7,8^. The fruits of snake gourd are frequently consumed when immature. As the fruits mature, the rind and flesh turn red, and the red flesh is used as a tomato substitute^2^. Snake gourd fruits can grow to 1.5 m in length, are serpentine in shape (hence the name snake gourd), and are used for ornamental purposes when they are mature. Their functional constituents include flavonoids, β-carotenoids, lycopene, and phenolic acids, which have beneficial pharmacological and therapeutic effects for humans^2,9–11^. For these reasons, snake gourd is a potentially economically important crop species with food, medicinal and ornamental value, and is worthy of further study and scientific research.

In the past decade, owing to the rapid advances in sequencing technology and bioinformatic algorithms, reference genomes of a number of cucurbit species have been assembled, including cucumber^12–17^, melon^18–20^, zucchini^21^, bottle gourd^22^, watermelon^23–25^, pumpkin^26,27^, wax gourd^28^, sponge gourd^29^, and bitter gourd^30^. However, no reference genome of snake gourd is available. Recent studies have shown that there is likely a common tetraploid ancestor of Cucurbitaceae species, and it has been inferred that cucurbits diverged from their common ancestor with grape 107–121 million years ago (Mya)^31^. Watermelon is believed to have differentiated from within the Cucurbitaceae family ~20.4 Mya and the divergence between melon and cucumber occurred 9.0–10.2 Mya^26,32,33^. However, the evolutionary history of the snake gourd genome remains largely unexplored.

In this study, we assembled the genome of snake gourd using Nanopore long reads combined with Hi-C chromatin interaction maps. Protein-coding genes and noncoding RNAs (ncRNAs) were predicted from the genome assembly, after which the predicted genes were functionally annotated. Phylogenetic and comparative genomic analyses indicated that snake gourd is closely related to sponge gourd. Transcriptome analysis revealed candidate genes that are involved in fruit texture, pigment accumulation, plant hormones, and resistance, and the expression profiles during fruit development and ripening, which may contribute to the unique characteristics of snake gourd fruits. The genome sequence presented in this study provides insights into both the structural characteristics of the snake gourd genome and the evolutionary relationship of snake gourd and related species and serves as a valuable resource for genomic research and comparative genomics within the Cucurbitaceae.

## Results

### Genome assembly, anchoring, and quality evaluation

**Fig. 1:**
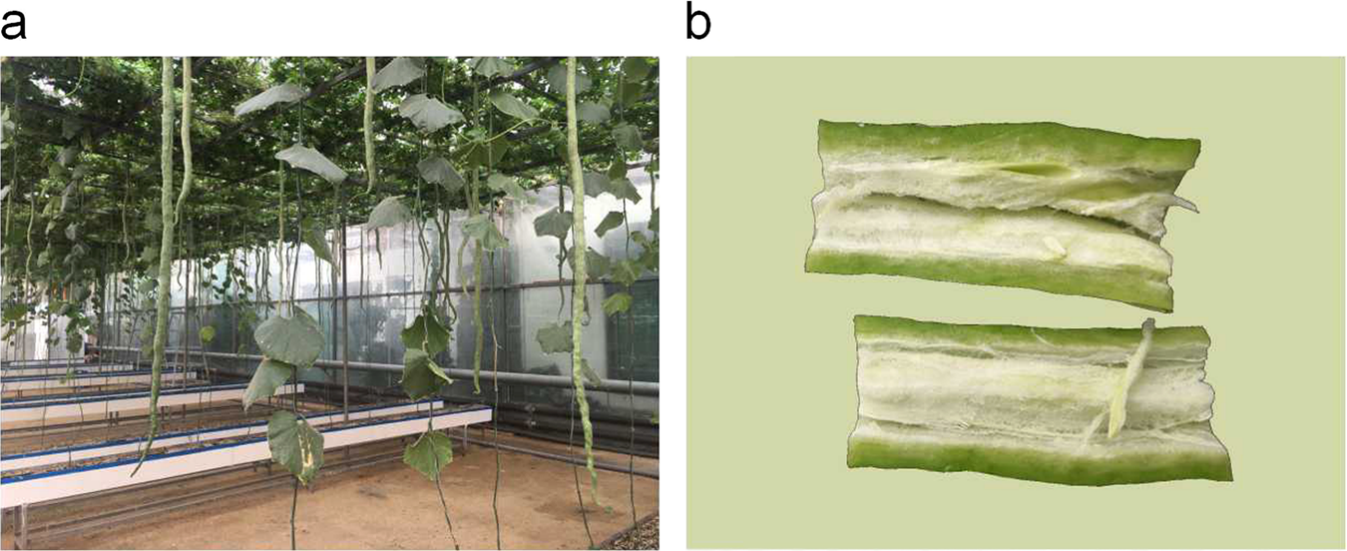
Panoramic and sectional view of snake gourd. **a** Snake gourd plants growing in the greenhouse of the Shouguang vegetable expo garden. **b** Longitudinal section of an immature snake gourd fruit

For this study, snake gourd plants were grown in a greenhouse, as shown in Fig. 1a. The immature fruits were greenish-white, long and slender (the length could reach 1.5–2 m). Figure 1b shows a longitudinal section of a snake gourd and the flesh of the immature fruit is white and tough. Two Illumina libraries with fragment sizes of ~350 bp were constructed for snake gourd and sequenced on the Illumina Novaseq 6000 sequencing platform (Illumina, 9885 Towne Centre Drive, San Diego, CA 92121, USA) in paired-end mode and with a read length of 150 bp. After cleaning, 65.5 Gb of high-quality data were obtained, representing a 63.6-fold depth of the snake gourd genome, which has an estimated size of 1.03 Gb based on the K-mer depth distribution analysis of the paired-end Illumina reads. The snake gourd genome is relatively large, highly homozygous, and estimated to contain 70.5% repetitive sequences, with a heterozygosity level of ~0.02% and a GC content of ~7.1%.

**Fig. 2:**
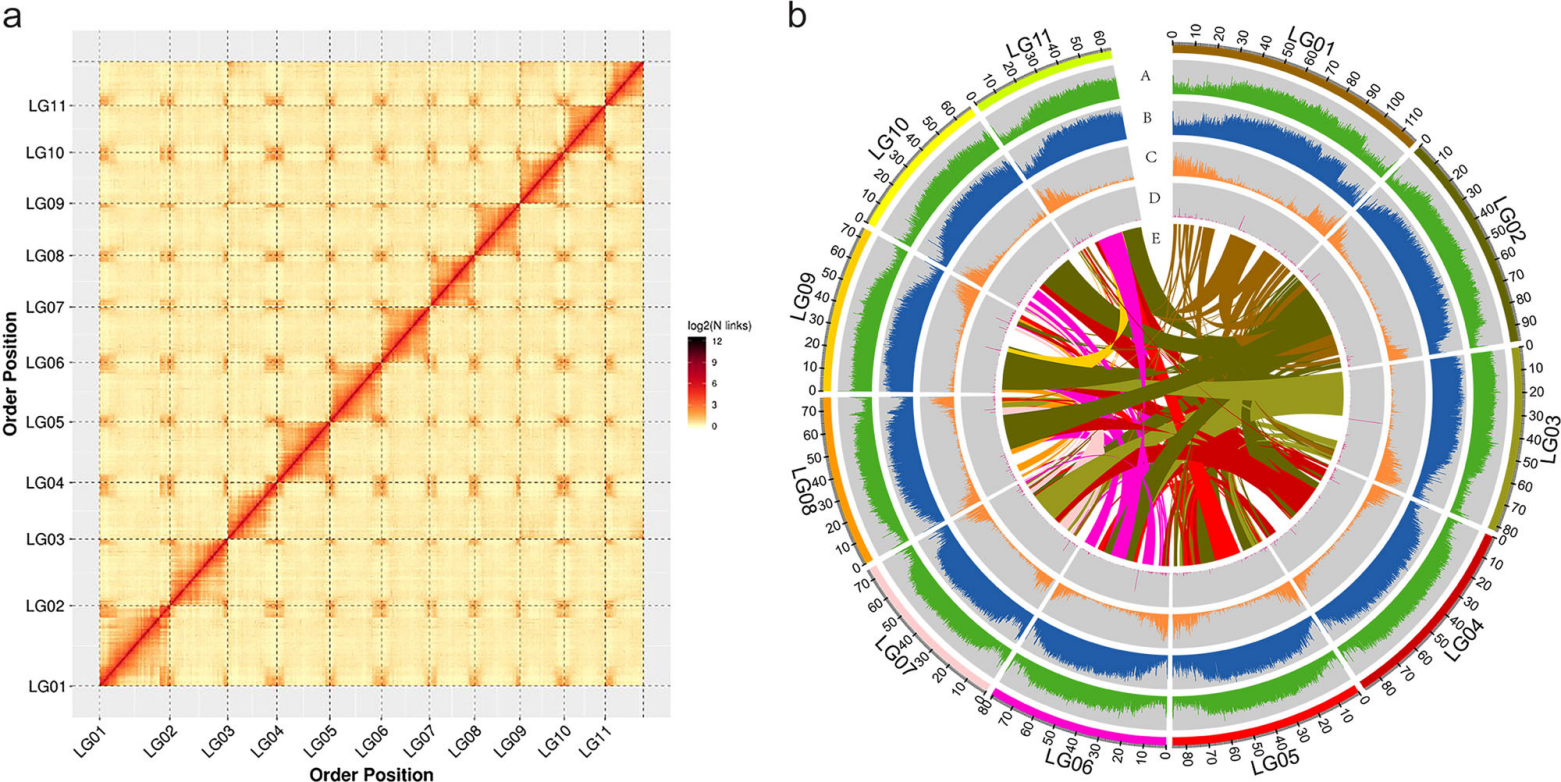
Snake gourd genome information. **a** Hi-C interaction heatmap of the snake gourd genome. The assembled genome was divided into 100 kb bins and valid interaction links of the Hi-C data were calculated between each pair of bins. The binary logarithm of the number of links in each bin was coded using colors ranging from light yellow to dark red, indicating the strength of Hi-C interaction links from low to high, respectively. LG01–LG11 represent the 11 chromosomes. **b** Landscape of the snake gourd genome. A: distribution of GC content (green); B: density of repeat sequences (blue); C: gene density (orange); D: noncoding RNA density (pink); E: syntenic blocks within the genome

A nanopore library was constructed and sequenced, generating a total of 135.9 Gb of raw data. After cleaning, the data were reduced to 111.8 Gb, representing ~108.5× the total data composing snake gourd genome, with an N50 read length of 32.7 kb. De novo assembly of the Nanopore reads resulted in an initial assembly of 919.8 Mb consisting of 167 contigs with an N50 length of 21.9 Mb (Table 1). The single-base error rate in the genome sequence using Nanopore sequencing technology was 0.00156%. After error correction with Illumina paired-end and Hi-C interaction maps, we obtained a final assembly that was 919.8 Mb in size comprising 202 contigs and 69 scaffolds, with a contig N50 of 20.11 Mb and a scaffold N50 of 82.12 Mb (Table 1). A chromosome interaction heatmap was constructed (Fig. 2a), which showed a pattern consistent with that of the main Hi-C genome assemblies and provided confidence in the pseudomolecule construction. Based on the Hi-C contact maps, a total of 197 sequences covering ~918.8 Mb (99.89% of the assembled genome) clustered into 11 groups that correspond to the 11 chromosomes of snake gourd (Fig. 2b), with the longest being 118.8 Mb and the shortest being 64.0 Mb (Supplementary Table 1 and Table 1).

**Table 1:** Statistics of the sequencing and assembly of the snake gourd genome

	Number	Size	N50 length
Nanopore reads	4,601,459	111.79 Gb	32.73 kb
De novo-assembled contigs (nanopore)	167	919.76 Mb	21.85 Mb
Final assembly (contigs)	202	919.76 Mb	20.11 Mb
Final assembly (scaffolds)	69	919.78 Mb	82.12 Mb
Chromosome-anchored contigs	197	918.75 Mb	–

To evaluate the quality of the assembly, we mapped the cleaned Illumina short reads to the assembled genome using BWA^34^, which had a mapping rate of 99.4%. Further evaluation using BUSCO (https://busco.ezlab.org/) with a database of 2,326 conserved core plant genes indicated that 95.7% of the core genes were found to be complete in the snake gourd genome assembly, including 93.5% that were single copies and 2.2% that were duplicated copies. In addition, 1.1% were found to be fragmented and only 3.2% were missing. Taken together, these results supported the integrity and high quality of the assembled snake gourd genome.

### Repeat sequence annotation and gene prediction

Based on the principles of structure prediction and de novo prediction, we constructed a repeat sequence database of the snake gourd genome, which was used to screen the assembly for repeat sequences. A total of 736.1 Mb (80.0%) of repetitive sequences were identified in the assembled genome. Among these repetitive sequences, long terminal repeat (LTR) retrotransposons were predominant, constituting 66.7% of the snake gourd genome assembly, with 45.6% belonging to the copia type and 19.8% belonging to the gypsy type (Supplementary Table 2).

Three prediction methods—an ab initio strategy, a homology-based strategy, and an RNA sequencing (RNA-seq) strategy—were used to predict protein-coding genes in the snake gourd genome and the predictions from these three methods were integrated using EVM^35^ v1.1.1 software (Supplementary Fig. 1). Ultimately, 22,874 protein-coding genes with a total length of 121.4 Mb were predicted in the genome (Table 2) and were functionally annotated, the annotations of which are shown in Supplementary Table 3. Of the predicted genes, a total of 22,129 (96.74%) were annotated by five functional databases (Table 2). In addition, we also identified 74 miRNAs, 294 rRNAs, 1,167 tRNAs, and 3,021 pseudogenes.

**Table 2:** Annotation statistics of the snake gourd genome assembly

Genome statistics	Number	Size	Percentage (%)
Total repetitive sequences	1,175,184	736.14 Mb	80.03
Pseudogenes	3,021	11.38 Mb	–
miRNAs	74	–	–
rRNAs	294	–	–
tRNAs	1,167	–	–
Total protein-coding genes	22,874	121.36 Mb	–
GO	11,969	–	52.33
KEGG	6,937	–	30.33
KOG	11,598	–	50.70
TrEMBL	22,073	–	96.50
Nr	22,116	–	96.69
In all databases	22,129	–	96.74

### Evolution of the snake gourd genome

**Fig. 3:**
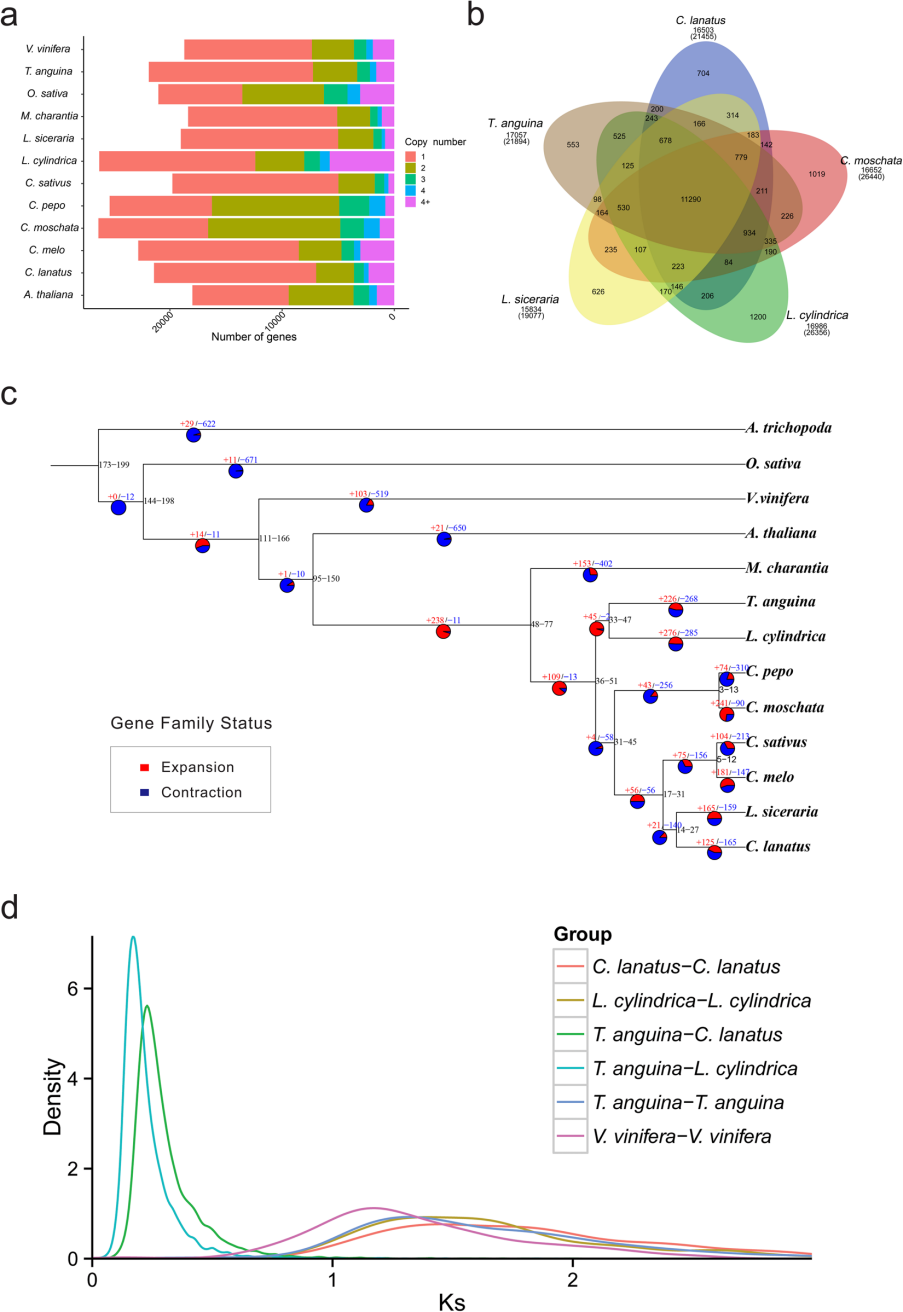
Gene family, phylogenetic analysis, and Ks distribution of snake gourd and other representative plant species. **a** Gene copy number distribution in snake gourd and 12 other plant species. **b** Venn diagram representing the clusters of gene families in snake gourd and four other related cucurbit species (sponge gourd, watermelon, bottle gourd, and pumpkin). **c** Phylogenetic tree of snake gourd and 12 other species based on a concatenated alignment of 970 single-copy protein sequences. The tree is rooted with *A. trichopoda* as the outgroup. CAFÉ-based estimates of gene family expansions and contractions. The red and blue numbers indicate expanded and contracted gene families, respectively. **d** Ks distribution in snake gourd and other representative plant species

To understand the evolution of the snake gourd genome, we collected genome sequences of representative plant species and performed a comparative genomic analysis with the genome sequence of snake gourd. Genes from 13 selected plant species, including 9 cucurbits (*T. anguina* L., *Luffa cylindrica* L., *Cucumis sativus* L., *Cucumis melo*, *Citrullus lanatus*, *Lagenaria siceraria*, *Cucurbita moschata*, *Cucurbita pepo*, and *Momordica charantia*), two rosid species (*Vitis vinifera* L. and *Arabidopsis thaliana*), one monocot (*Oryza sativa*), and one in the basal lineage of angiosperms (*Amborella trichopoda*), clustered into 34,827 gene families. A total of 17,057 gene families were identified in snake gourd, 125 of which (comprising 451 genes) were specific to the snake gourd genome (Supplementary Table 4). Clustering analysis revealed a total of 14,148 single-copy genes in snake gourd, accounting for 61.9% of the predicted genes, which is similar to that in other cucurbit species, such as *L. cylindrica*, *C. sativus*, *C. melo*, *C. lanatus*, *L. siceraria*, and *M. charantia*, in which no recent whole-genome duplication (WGD) events occurred, but is substantially higher than that in *C. moschata* and *C. pepo*, whose genomes underwent a recent WGD event (Fig. 3a). The clustering of gene families in snake gourd and several other cucurbit species, including sponge gourd, watermelon, bottle gourd, and pumpkin, is shown in Fig. 3b and indicates that the number of snake gourd gene families (17,057) was most similar to the number of sponge gourd gene families (16,986).

In addition, Gene Ontology (GO) and Kyoto Encyclopedia of Genes and Genomes (KEGG) enrichment analyses were performed for gene families specific to snake gourd. GO analysis showed that these families were enriched in genes involved in metabolic processes, cellular processes, and biological regulation or encoded products that have binding, nucleic acid binding, DNA binding, and catalytic activity function (Supplementary Fig. 2b). KEGG pathway analysis indicated that genes related to flavonoid biosynthesis, regulation of autophagy, fatty acid degradation, etc., were enriched in the snake gourd-specific families (Supplementary Fig. 2a). These included two genes (*EVM0012390.1* and *EVM0020640.1*) encoding flavonol synthase/flavanone 3-hydroxylase in the flavonoid biosynthetic pathway, two autophagy-related genes associated with the regulation of the autophagy pathway, and two genes encoding alcohol dehydrogenases (ADHs) (*EVM0001187.1* and *EVM0017122.1*), which are involved in fatty acid degradation, tyrosine metabolism, α-linolenic acid metabolism, and glycolysis/gluconeogenesis. Furthermore, a number of other genes were identified that are known to participate in fruit ripening, such as those encoding cell wall-associated hydrolase, phenylalanine ammonia-lyase (PAL) 1, malonate–CoA ligase, and aquaporin PIP2-2. Together, these specific genes and gene families may contribute to the unique fruit features of snake gourd (Table 3).

**Table 3:** Select gene annotations for specific gene families in snake gourd

Gene ID	KEGG pathway	Nr annotation
EVM0001187.1	Fatty acid degradation, tyrosine metabolism, α-linolenic acid metabolism, glycolysis/gluconeogenesis	*alcohol dehydrogenase-like*
EVM0017122.1	Fatty acid degradation, tyrosine metabolism, α-linolenic acid metabolism, glycolysis/gluconeogenesis	*alcohol dehydrogenase-like*
EVM0008214.1	Regulation of autophagy	*autophagy-related protein 8C-like*
EVM0014225.1	Regulation of autophagy	*autophagy-related protein 8C-like isoform X4*
EVM0012390.1	Flavonoid biosynthesis	*flavonol synthase/flavanone 3-hydroxylase*
EVM0020640.1	Flavonoid biosynthesis	*flavonol synthase/flavanone 3-hydroxylase*
EVM0002095.2	Valine, leucine, and isoleucine degradation	*malonate–CoA ligase-like*
EVM0010593.1	Valine, leucine, and isoleucine degradation	*malonate–CoA ligase-like*
EVM0011798.1	Phenylalanine metabolism, phenylpropanoid biosynthesis	*phenylalanine ammonia-lyase 1-like*
EVM0020582.1	Phenylalanine metabolism, phenylpropanoid biosynthesis	*phenylalanine ammonia-lyase-like*
EVM0003408.1		*Cell wall-associated hydrolase*, *partial*
EVM0005117.1		*aquaporin PIP2-2-like*
EVM0021260.1		*aquaporin PIP2-2-like*

Based on 970 single-copy genes in snake gourd and in 12 other plant species, a phylogenetic tree was constructed. The phylogenetic tree showed that, of all the Cucurbitaceae species examined, snake gourd is most closely related to sponge gourd (Fig. 3c). Snake gourd and sponge gourd diverged from their common ancestor approximately 33–47 Mya after the divergence from the common ancestor together with bitter gourd (44–87 Mya) but before the divergence between watermelon and bottle gourd (14–27 Mya) (Fig. 3c). In addition, we performed a comparative analysis of the evolution of gene families in the 13 plant species. In snake gourd, 226 gene families comprising 1406 genes exhibited significant expansion (*p* < 0.01) relative to those of the last common ancestor, whereas 268 gene families showed contraction (Fig. 3c). KEGG and GO functional analyses showed that most of the members of the expanded snake gourd gene families are involved in metabolic processes, cellular processes, cell catalytic activity, binding, flavonoid biosynthesis, regulation of autophagy, fatty acid degradation, and tyrosine metabolism (Supplementary Fig. 3), and included genes encoding cellulose synthase, wall-associated receptor kinase, exopolygalacturonase, polygalacturonase (PG), lipoxygenase, linoleate 9S-lipoxygenase 6, terpene synthase, (−)-germacrene d-synthase, (+)-γ-cadinene synthase, myrcene synthase, 1-aminocyclopropane-1-carboxylate oxidase (ACO), auxin-induced protein 22D, 23 kDa jasmonate-induced protein, anthocyanidin 3-*O*-glucosyltransferase, and flavonoid hydroxylase. These genes are involved in cell wall biosynthesis and degradation, flavor and aromatic compound generation, phytohormone synthesis and signal transduction, and flavonoid biosynthesis. Other gene families involved in plant defense and response to pathogens were also found to be expanded in snake gourd, such as those whose members encode UDP-glucosyltransferase, salicylate carboxymethyltransferase, tobacco mosaic virus (TMV) resistance protein N, and other disease resistance proteins.

WGD events have occurred widely in angiosperms and many plant species have experienced genome duplications in their evolutionary history, which are of great significance in understanding speciation, genome evolution, and gene neofunctionalization^36^. However, our in-depth genomic analysis indicated that no recent WGD events have occurred in the snake gourd genome, which is consistent with the findings of a recent report^37^. The Ks distribution among these species suggested that snake gourd diverged from sponge gourd ~42 Mya and from watermelon ~47 Mya (Fig. 3d). A peak centering on a Ks of ~1.32 was observed between snake gourd paralogous pairs (Fig. 3d), which corresponds to the ancient whole-genome triplication (the γ event) shared by all core eudicots^31^. In addition, genome collinearity analysis between snake gourd, sponge gourd, and watermelon (Supplementary Fig. 4) showed a high degree of gene-order conservation with the snake gourd genome.

### Transcriptomes at different ripening stages of snake gourd fruits

**Fig. 4:**
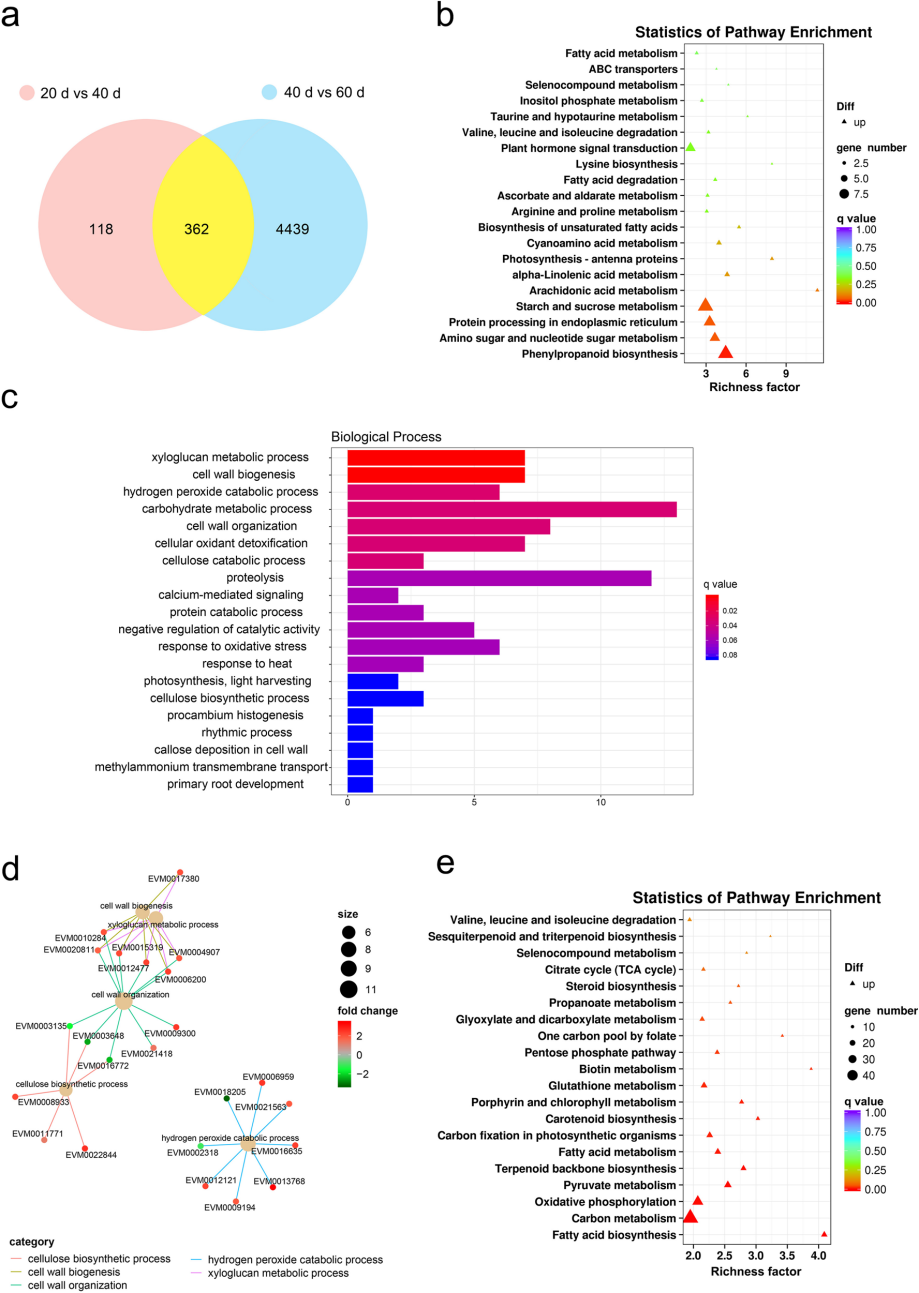
Differentially expressed genes (DEGs) at different stages of snake gourd fruit and changes in transcription during fruit development and ripening. **a** Venn diagram representing the number of DEGs between the 20 d vs. 40 d and 40 d vs. 60 d groups; 362 DEGs overlapped between the two groups. **b** Upregulated KEGG pathways in the 20 d vs. 40 d group comparison. **c** Upregulated GO terms related to biological processes in the 20 d vs. 40 d group comparison. **d** Netplot of significantly enriched GO terms in the 20 d vs. 40 d group comparison. **e** Upregulated KEGG pathways in the 40 d vs. 60 d group comparison

To identify potential candidate genes related to fruit characteristics and quality during fruit development and ripening, transcriptome analysis was used to study changes in differentially expressed genes (DEGs) in the fruits at different stages. The results showed that there were 362 common DEGs in the development and ripening stages (20 d vs. 40 d and 40 d vs. 60 d) (Fig. 4a). Among them, five genes related to disease resistance and defense responses overlapped between the two groups, *leucine-rich repeat receptor-like protein kinase TDR*, *LRR receptor-like serine/threonine-protein kinase FEI 1*, *TMV resistance protein N*, *leucine-rich repeat extensin-like protein 6*, and *respiratory burst oxidase homolog protein E*, all of which participate in the plant–pathogen interaction pathway (Supplementary Table 5). These genes were upregulated at 20 d vs. 40 d and downregulated at 40 d vs. 60 d, which may be related to changes in fruit resistance during ripening.

During the period of fruit development, a total of 480 genes, including 356 (74%) upregulated and 124 (26%) downregulated genes, were differentially expressed in the 20 d vs. 40 d comparison. KEGG pathway analysis of the upregulated genes showed that they were mainly enriched in the pathways involved in phenylpropanoid biosynthesis, amino sugar and nucleotide sugar metabolism, protein processing in the endoplasmic reticulum and starch and sucrose metabolism (Fig. 4b). The expression of several peroxidase genes, such as *peroxidase 31* (increase of 3.4-fold), *peroxidase 55-like* (increase of 4.0-fold), *peroxidase 5* (increase of 6.4-fold), *peroxidase 64-like* (increase of 8.8-fold), *peroxidase 2-like* (increase of 11.6-fold), *peroxidase 21-like* (increase of 10.5-fold), and *probable mannitol dehydrogenase* (increase of 2.1-fold), markedly changed in snake gourd fruit (Supplementary Table 5). Of the upregulated GO terms, the most enriched categories related to biological processes included the xyloglucan metabolic process, cell wall biogenesis, the carbohydrate metabolic process, and cell wall organization (Fig. 4c). The corresponding gene networks associated with significantly abundant GO items in the 20 d vs. 40 d group are shown in Fig. 4d. Many enzymes related to cell wall metabolism were identified as being upregulated, including β-glucosidase (BGLU) 18-like, β-galactosidase (GAL) 5-like/10, pectinesterase-like (PE), expansin-A4/A10-like (EXP), endoglucanase (EG) 10/11/17-like, glucan endo-1,3-β-d-glucosidase, and glucan endo-1,3-β-glucosidase 13-like but not cellulose synthase-like protein H1. In addition, increased expression of plant hormone-related genes was also found, including *abscisic acid receptor PYR1/PYL4-like*, *auxin-induced protein 22D-like*, and *auxin-responsive protein IAA16/IAA21-like/IAA29-like*. It is generally known that the phytohormone auxin can regulate cell growth and induce tracheary element differentiation^38^.

We identified 4801 genes that were differentially expressed by comparing the transcriptome at 60 d with that at 40 d. A total of 1957 (41%) genes were upregulated and 2844 (59%) genes were downregulated. In specific gene families of snake gourd, five genes were found to be differentially expressed: *PAL*, *cysteine protein inhibitor 1-like*, *MLP-like protein 329*, *poly transporter 5-like*, and *PAL 3*. Their expression profiles were found to be downregulated after 60 d compared with 40 d. The results of the KEGG pathway analysis of the upregulated genes suggested that the most enriched pathways were involved in fatty acid biosynthesis, carbon metabolism, pyruvate metabolism, and carotenoid biosynthesis (Fig. 4e). Among the 15 genes involved in carotenoid synthesis, 10 were upregulated, including *phytoene synthase* (*PSY*) (increase of 1.7-fold), *15-cis-phytoene desaturase* (increase of 4.6-fold), *15-cis-zeta-carotene isomerase* (increase of 5.1-fold), *zeta-carotene desaturase* (*ZDS*) (increase of 19.0-fold), *prolycopene isomerase* (*CRTISO*) (increase of 25-fold), and *β-carotene 3-hydroxylase 1* (increase of 40.0-fold), and 5 were downregulated, including *lycopene epsilon cyclase*, *lycopene β-cyclase*, *probable 9-cis-epoxycarotenoid dioxygenase NCED5*, and *abscisic acid 8-hydroxylase 4-like*. Moreover, 11 genes related to the accumulation of flavonoids and anthocyanidins in the fruits were also found, including *anthocyanidin 3-O-glucosyltransferase 2-like*, *flavonoid 3-monooxygenase-like*, *flavonoid 3*,*5-methyltransferase-like*, *flavonol synthase/flavanone 3-hydroxylase-like*, and *isoflavone reductase-like protein*, which exhibited biphasic expression patterns. *PAL* is involved in the phenylalanine metabolism pathway and functions prior to lignin synthesis, and the expression of many *peroxidases* related to enzymes associated with lignification, the cell wall, auxin catabolism, defense against pathogens, and scavenging of reactive oxygen species is downregulated. In addition, the expression levels of numerous genes related to plant hormones synthesis and signal transduction were significantly altered: the levels of *ACO homolog 1/3-like*, *ethylene receptor 2-like*, *ethylene response sensor 1*, the *serine/threonine-protein kinase CTR1*, *ethylene insensitive 3-like 3 protein*, *auxin response factor 18* (*ARF*), *auxin-responsive protein SAUR71-like*, *gibberellin-regulated protein 1-like* (*GRP*), and *indole-3-acetic acid-amido synthetase GH3.17* were upregulated, and the expression levels of others, such as *ACO1/3*, *ethylene-responsive transcription factor 1B-like* (*ERF*), *indole-3-acetic acid-amido synthetase GH3.10/GH3.6-like*, *auxin-induced protein 15A/22D/22B-like*, *ARF 4/9/18*, *auxin-responsive protein IAA/SAUR50-like*, *auxin transporter-like protein 1/2*, the *jasmonic acid-amido synthetase JAR1*, *abscisic acid receptor PYR1-like*, *GRP 9/4-like*, and the *AP2-like ethylene-responsive transcription factor ANT*, were downregulated. Furthermore, the mRNAs for some enzymes related to fruit cell wall structure also changed significantly, with *PE*, *PE 3-like*, *pectate lyase 5/8/12/18* (*PL*), *pectin acetylesterase 8-like*, *PG-like*, *PG At1g48100-like*, *PG QRT3-like*, *GAL 3/10/5-like*, *BGLU 18/44-like*, *glucan endo-1*,*3-β-glucosidase*, *expansin* (*EXP*), *EG 6/10/11/17/24-like*, and *cellulose synthase-like protein G3* downregulated while others such as *β-d-xylosidase1-like*, *GAL 13-like*, *GAL 16*, *GAL-like*, *cellulose synthase-like protein E1/H1*, and *EG 9-like* were downregulated. These changes in mRNAs of cell wall enzymes may be related to fruit texture changes, as the fruits were softer after 60 d compared with 40 d.

**Fig. 5:**
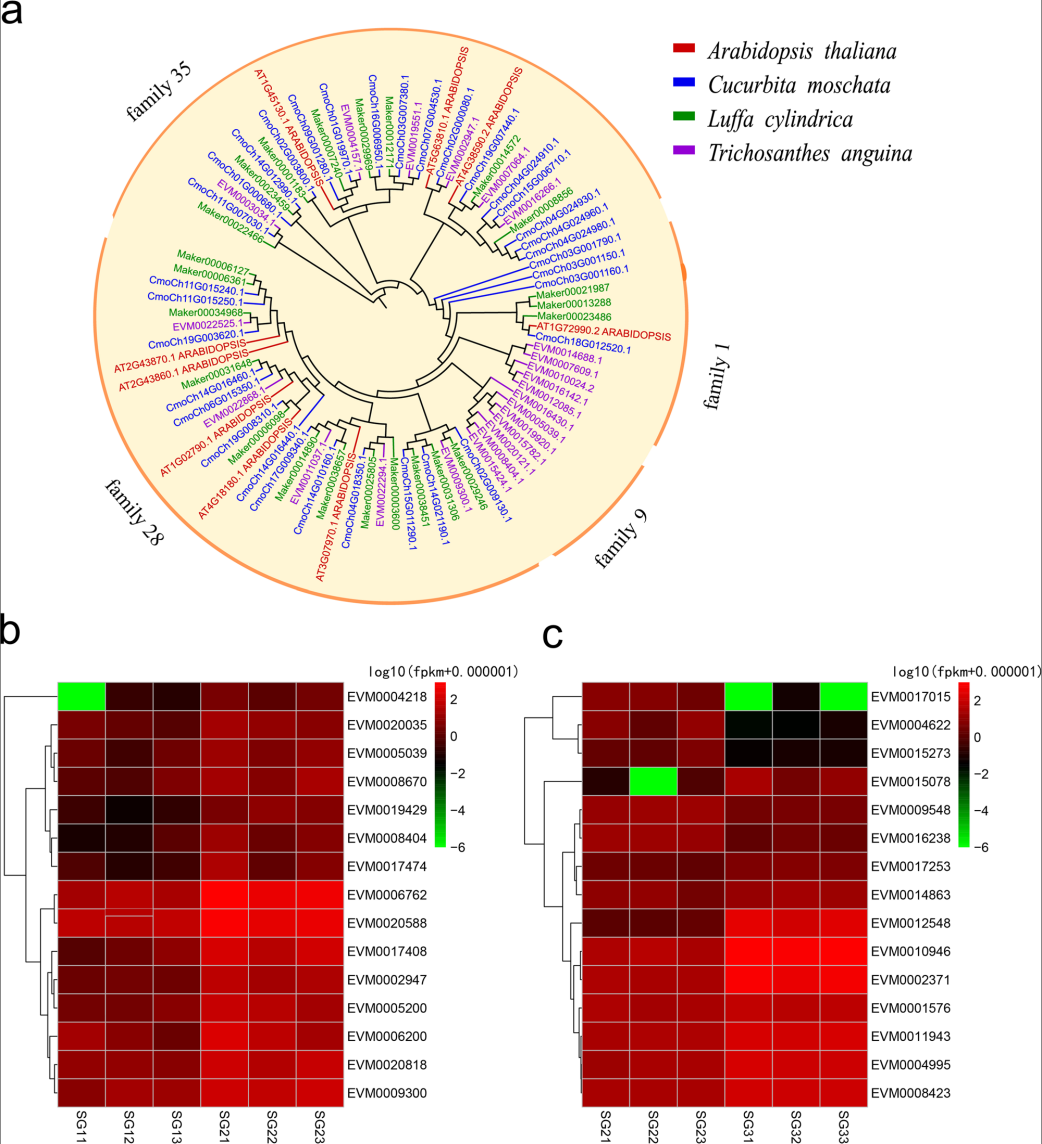
Phylogenetic tree of glycosyl hydrolase families (GHs) and a heatmap, with replicates, of differentially expressed genes (DEGs) at different stages in snake gourd fruits. **a** Phylogenetic tree of glycosyl hydrolase families among snake gourd, sponge gourd, *Arabidopsis*, and pumpkin. **b** Heatmap showing the expression of fruit texture-related genes at the 20 d (SG1) and 40 d (SG2) stages. **c** Heatmap showing the expression of fruit pigment accumulation-related genes at the 40 d (SG2) and 60 d (SG3) stages. The red and green colors indicate high and low expression levels, respectively

We focused our subsequent analysis on cell wall-related gene families whose members are responsible for fruit texture in snake gourd. The glycosyl hydrolase family (GH) related to cell wall structure was selected to construct a phylogenetic tree. The GH families were divided into four subfamilies: GH-1, GH-9, GH-28, and GH-35. The members of the GH-28 and GH-35 subfamilies encode the majority of GH proteins. The genes belonging to the GH-28 and GH-35 subfamilies in snake gourd showed the closest phylogenetic relationships with those of pumpkin and sponge gourd (Fig. 5a). In the snake gourd genome, the GH-1 subfamily contained five members, but only one, one, and three proteins belonged to the GH-1 subfamily in *Arabidopsis*, pumpkin, and sponge gourd, respectively; moreover, there were no proteins that clustered within the GH-9 subfamily in these three plant species (Fig. 5a). Based on the transcriptome data, we further analyzed the expression of 15 DEGs related to cell wall modification and pigment accumulation in the 20 d vs. 40 d (SG1 vs. SG2) and 40 d vs. 60 d (SG2 vs. SG3) group comparisons, respectively. A heatmap of these DEGs showed the gene expression profiles at different stages (Fig. 5b, c). In the 20 d vs. 40 d group comparison (SG1 vs. SG2), numerous members of the GH family were highly expressed at this fruit development stage, with the exception of PE (EVM0004218), and the results of the three replications were similar (Fig. 5c). In the 40 d vs. 60 d group comparison (SG2 vs. SG3), ZDS (EVM0002371), prolycopene isomerase (EVM0010946), and 9-*cis*-epoxycarotenoid dioxygenase NCED2 (EVM0012548) had much higher expression in SG3, whereas the expression levels of abscisic acid 8-hydroxylase 4-like (EVM0017015 and EVM0004622) and 9-*cis*-epoxycarotenoid dioxygenase NCED5 (EVM0015273) decreased during fruit maturation (Fig. 5c).

## Discussion

Snake gourd is an excellent vegetable species that is popular for both its unique flavor and its nutritional, health, medicinal and ornamental value and has high utilization potential^2^. However, research on this species is limited^3^, and related molecular-level studies are scarce. We aimed to sequence, assemble, annotate, and comparatively analyze the snake gourd genome to provide a basis for future investigations and improvement of this species. This is the first report of a high-quality genome for snake gourd, which has one of the largest genomes of Cucurbitaceae species sequenced to date.

The assembled genome size is ~919.76 Mb, which is close to that of wax gourd (913 Mb) but larger than those of other Cucurbitaceae species, such as watermelon, melon, pumpkin, bottle gourd, cucumber, zucchini, bitter gourd (200–400 Mb), and sponge gourd (669 Mb)^17–30^ (Table 4). We predicted 22,874 protein-coding genes, which is similar to the number of genes present in melon^19^, slightly more than the number in bottle gourd^22^, but less than the number in the eleven other Cucurbitaceae species examined (Table 4). Repetitive sequences such as transposable elements play crucial roles in genome evolution, chromosome rearrangement, and gene regulation^39^ but have presented a major challenge for producing high-quality genome assemblies. We found that 80.0% of the snake gourd genome is composed of repeated sequences, with LTRs accounting for 66.7% of the genome, which is quite high compared with the percentage of repeat content in other Cucurbitaceae genomes. BUSCO (https://busco.ezlab.org/)-based assessment revealed that the snake gourd assembly is more complete than or comparable to other reported cucurbit reference genomes (Table 4) and provides valuable information about evolutionary events and gene family expansion in snake gourd; in addition, this assembly serves as a useful resource for comparative genomic studies in Cucurbitaceae.

**Table 4:** Comparison of the snake gourd genome assembly and other Cucurbitaceae genome assemblies

	Genome size	Contig N50	Scaffold N50	Complete BUSCOs	Repetitive sequences	LTRs	Protein-coding genes
Snake gourd	919.76 Mb	20.11 Mb	82.12 Mb	95.38%	80.03%	66.74%	22,874
Watermelon^23^	353.5 Mb	26.38 kb	2.38 Mb	–	45.20%	–	23,440
Watermelon^24^	365.1 Mb	2.3 Mb	21.9 Mb	–	55.55%	–	22,596
Melon^19^	386 Mb	2.86 Mb	–	92.78%	49.80%	30.16%	22,924
Melon^18^	375 Mb	18.2 kb	4.68 Mb	–	19.70%	12.80%	27,427
Sponge gourd^29^	669 Mb	5 Mb	53 Mb	91.60%	62.18%	58.32%	31,661
Bottle gourd^22^	313.4 Mb	28.3 kb	8.7 Mb	95.40%	46.90%	39.80%	22,472
Silver-seed gourd^27^	228.8 Mb	463.4 kb	620.88 kb	93.20%	34.10%	49.09%	28,298
Cucumber^17^	226.2 Mb	8.9 Mb	11.5 Mb	–	36.43%	–	24,317
Zucchini^21^	263 Mb	110 kb	1.8 Mb	92.10%	37.80%	50.70%	27,870
Bitter gourd^30^	285.5 Mb	–	1.1 Mb	–	15.30%	9.90%	45,859
*C. maxima*^26^	271.4 Mb	40.7 kb	3.7 Mb	95.50%	40.30%	69.90%	32,076
*C. moschata*^26^	269.9 Mb	40.5 kb	4.0 Mb	95.90%	40.60%	62.90%	32,205
Wax gourd^28^	913 Mb	68.5 kb	3.4 Mb	91.00%	75.50%	57.70%	27,467

In specific gene families of snake gourd, a number of genes have been found to potentially participate in fruit ripening, such as several genes encoding autophagy-related proteins, ADHs, and the aquaporin PIP2-2. Autophagy is involved in plant processes such as floret ripening (in wheat)^40^, root tip cell growth and differentiation^41^, and chloroplast degradation in senescing leaves^42^. The autophagy pathway has also been reported to be involved in plant innate immunity^43^ and responses to a variety of abiotic stresses^44^, and in assisting plants in surviving nutrient-limitation stress^45^. ADH enzymes play multiple roles in anaerobic fermentation, aerobic fermentation, and the production of scents that discourage predation, attract pollinators, and facilitate seed dispersal^46^. In petunia, both the *ADH2* and *ADH3* genes act in the lipoxygenase pathway to produce floral scents^47^ and flavor volatiles in ripening fruits. A previous study reported a strawberry fruit-specific aquaporin, FaPIP1;1, which showed an expression profile associated with fruit ripening^48^. Further study indicated that the FaPIP aquaporins showed an expression pattern associated with fruit firmness^48^. In addition, several specific genes were found to be differentially expressed between 40 d and 60 d, which may contribute to the unique snake gourd fruit features.

Between 20 d and 40 d, the snake gourd fruits became longer and harder, and we detected increased transcript levels of genes related to cell division, cell expansion, fruit growth, and protection of the fruit surface^49^. We identified transcripts for highly expressed cell wall-modifying genes similar to those expressed in expanding cucumber^50,51^, tomato^52^, melon^53^, and watermelon^54^ fruits, such as *EXP*s, *endo-1*,*2-B-glucanase*, *BGLU*s, *PL*s, and *pectin methylesterases*. Major increases in transcripts for mRNAs encoding enzymes related to cell wall metabolism were detected, including *GAL 10* (increase of 14.3-fold), *PE-like* (increase of 21.6-fold), *EG 17-like* (increase of 21.2-fold), *expansin-A10-like* (increase of 27.1-fold), *UDP-glucuronate 4-epimerase 1-like* (increase of 29.8-fold), and *PE 53* (increase of 30.4-fold), which may be involved in the production of longer and harder fruits. We also detected the upregulated expression of auxin-related genes. These genes may be involved in fruit elongation, as many studies have shown that phytohormones, especially auxin and gibberellins, are related to fruit growth and changes in fruit shape^55^. These results are consistent with those of previous studies that found that three homologs of auxin-related genes were differentially expressed in developing watermelon fruits^54^. In addition, we found an increase in resistance (R) gene transcripts, such as those of *TMV resistance protein N*, *leucine-rich repeat extensin-like protein 3*, and *LRR receptor-like serine/threonine-protein kinase FEI 1*. Studies have shown that younger and smaller fruits are more susceptible to infection than older and larger fruits^56^, and changes in the expression of resistance genes may explain the stronger resistance of larger fruits compared with smaller ones. These genes mainly regulate the resistance of plants to pathogens and insects, and in cucumber, eight DEGs have been identified that may be associated with aphid resistance^57^.

Transcripts for several enzymes mentioned above related to cell wall metabolism were found to be downregulated during ripening from 40 to 60 d, which may be the cause of fruit softening at 60 d. The expression levels of these enzymes are different among various fruits and at different fruit developmental stages. Studies on watermelon have shown that there are differences in the expression of genes related to cell wall components in two kinds of flesh with different textures^58^, and the key genes involved in the regulation of flesh texture were differentially expressed in cultivated and wild watermelon^59^. In addition, the genes involved in the upstream and downstream steps of the carotenoid biosynthesis or degradation pathway were upregulated and downregulated, respectively. These changes may contribute to the increased carotenoid accumulation, making the fruit turn orange after ripening. This is supported by research on carotenoid accumulation and related gene expression during squash fruit development^60^.

## Materials and methods

### Snake gourd sample collection and genome sequencing

Young fresh leaf samples were collected from snake gourd plants grown in the greenhouse of the Shouguang vegetable expo garden. Genomic DNA was extracted from young leaves and used to construct two Illumina DNA libraries whose fragment size was ~350 bp, according to the standard protocols provided by the Illumina company. The libraries were sequenced on an Illumina HiSeq X platform in paired-end mode and with read length of 150 bp. The sequence data were evaluated (GC distribution statistics and quality value assessment) and filtered to obtain high-quality clean reads, which were subsequently used for estimation of the genome size, GC content, heterozygosity level, and postassembly error correction and evaluation. The raw sequencing reads were processed for quality control and for the removal of adapter and low-quality sequences using a custom Perl script. After trimming, reads with lengths less than 100 bp or a Q30 value < 85% were discarded.

A Nanopore library was constructed from high-molecular-weight DNA using a Ligation Sequencing Kit (SQK-LSK109) and sequenced on a PromethION R9 flow cell with a PromethION Flow Cell Priming Kit (EXP-FLP001.PRO.6) (Oxford Nanopore Technologies, UK) according to the manufacturer’s instructions. The Oxford Nanopore reads were self-corrected using Canu^61^ and the corrected reads were assembled into contigs using SMARTdenovo (https://github.com/ruanjue/smartdenovo). The assembled contigs were further polished using the Racon^62^ and Pilon^63^ programs in conjunction with cleaned Illumina reads.

We also constructed Hi-C fragment libraries (insert size of 300–700 bp) and sequenced them using the Illumina platform. The raw Hi-C reads were processed to trim adapter sequences and to remove low-quality reads. The cleaned Hi-C reads were first truncated at the putative Hi-C junctions, and then the resulting trimmed reads were aligned to the genome assembly with BWA^34^ (version 0.7.10-r789). Only uniquely aligned paired-end reads with a mapping quality greater than 20 were used for further analysis. Invalid read pairs including dangling-end and self-cycle, religation and dumped products were filtered by HiC-Pro^64^ v2.8.1. Approximately 75% of the uniquely mapped read pairs were valid interaction pairs and were used for correction of the assembled contigs, which were then clustered, ordered, and oriented onto chromosomes by LACHESIS^65^ with the parameters “CLUSTER_MIN_RE_SITES, 52; CLUSTER_MAX_LINK_DENSITY, 2; ORDER_MIN_N_RES_IN_TRUN, 51; ORDER_MIN_N_RES_IN_SHREDS, 50.”

### Gene prediction and functional annotation

A repetitive sequence database of the snake gourd genome was constructed using LTR_FINDER^66^ and RepeatScout^67^. Repeat sequences in the database were classified using PASTEClassifier^68^ and then merged together with the repeats in RepBase^69^ to construct the final repetitive sequence database. Repetitive sequences of the snake gourd genome were predicted using RepeatMasker^70^ based on the constructed repetitive sequence database.

Protein-coding genes were predicted from the snake gourd genome using three different strategies: ab initio prediction, homology-based prediction, and transcript-based prediction. We used GENSCAN^71^, Augustus^72^ v2.4, GlimmerHMM^73^ v3.0.4, GeneID^74^ v1.4, and SNAP^75^ (version 2006-07-28) for ab initio predictions, and GeMoMa^76^ v1.3.1 was used for homology-based predictions using protein sequences from *A. thaliana*, *C. lanatus*, *C. sativus*, and *L. cylindrica*. For transcript-based predictions, a mixture of four tissues (root, stem, leaf and fruit tissues) of snake gourd was used to construct an Illumina RNA-Seq library, which was subsequently subjected to PE (2 × 150 bp) sequencing on an Illumina HiSeq X Ten platform (Illumina, San Diego, CA, USA). After discarding the reads with low-quality bases, adapter sequences, and duplicated sequences, the retained high-quality clean reads were mapped to the snake gourd genome assembly using HISAT2^77^ v2.0.4 and, based on the mapping results, the reads were assembled into transcripts using StringTie^78^ v1.2.3, TransDecoder v2.0 (http://transdecoder.github.io) and GeneMarkS-T^79^ v5.1; the assembled transcripts were subsequently used for gene prediction. RNA-Seq reads were also de novo-assembled using Trinity^80^ and PASA^81^ v2.0.2 was used for gene prediction from these de novo*-*assembled transcripts. Finally, EVM^35^ v1.1.1 was used to integrate the prediction results obtained by the above three methods. In addition, we also predicted different ncRNAs. MicroRNA and rRNA sequences were predicted based on the comparison of the snake gourd genome to the content of the Rfam^82^ database via BLASTn and tRNA sequences were predicted using tRNAscan-SE^83^. Pseudogene prediction was also performed. Using the predicted protein sequence, through a BLAST^84^ comparison, we identified possible homologous gene sequences in the genome and then used GeneWise^85^ to identify premature termination codons and frameshift mutations in the gene sequences to identify pseudogenes.

The protein sequences of the snake gourd genes were compared to the content of the NCBI nr^86^, KOG^87^, GO^88^, KEGG^89^, and TrEMBL^90^ databases using BLAST^91^ v2.2.31, with an *E*-value cutoff of 1 × 10^−5^, and functional annotations of the snake gourd genes were derived from the homologous sequences in these databases.

### Comparative genomic analysis

The protein sequences from snake gourd and 12 other representative plant species were clustered into orthologous groups using OrthoFinder^92^ v2.3.7. The obtained orthologous groups (gene families) were annotated using the PANTHER v15 database^93^. GO and KEGG enrichment analyses of gene families unique to snake gourd were ultimately performed using ClusterProfile^94^ v3.14.0.

By the use of 970 single-copy protein sequences, an evolutionary tree was constructed using the maximum likelihood method implemented in IQ-TREE^95^ v1.6.11, with *A. trichopoda* as the root and the number of bootstraps set to 1000. We then used MCMCTee software in the PAML package^96^ v4.9i to calculate the divergence time. The number of iterations of the Markov chain included a burn-in number of 700,000, a sampfreq of 30, and an nSample of 6,000,000.

Using the results of the evolutionary tree with differentiation time and gene family clustering by CAFE^97^ v4.2, we estimated the number of gene family members of each branch’s ancestor via a birth mortality model, which was used to predict the contraction and expansion of gene families of the snake gourd relative to its ancestors. We determined whether the expansion or contraction was significant using a *P*-value cutoff of 0.05. The expanded and contracted gene families identified in snake gourd were annotated with PANTHER, and GO and KEGG enrichment analyses on these families were performed with ClusterProfile.

### Collinearity and WGD analyses

Diamond^98^ v0.9.29.130 was used to compare the protein sequences of two species and determine similar gene pairs (*E*-value < 1 × 10^−5^, C score > 0.5). Based on the diamond results, collinear blocks between the genomes of the two species were identified using MCScanX^99^.

WGD events were determined based on the distribution of the synonymous substitution rate (*Ks*) and fourfold degenerate (4DTv) sites of paralogous genes, which were calculated using WGD software^100^ in conjunction with a Perl script (https://github.com/JinfengChen/Scripts), respectively.

### Transcriptome

Fresh snake gourd fruit samples at 20, 40, and 60 d were collected for transcriptome analysis. cDNA libraries were obtained by PCR enrichment. After the library was checked for quality by quantitative PCR, the Illumina platform was used for sequencing. The data were cleaned by removing low-quality sequences and those containing adapter reads and used for sequence alignment with the specified reference genome. The transcriptome was assembled using StringTie^78^. Differential expression analysis was performed between the different sample groups. Three replicates were used for each sample and DESeq2^101^ was used for differential expression analysis between sample groups to obtain the DEG sets between two biological samples. During the detection of DEGs, a fold change > 2 and a false discovery rate (FDR) < 0.01 were used. As a screening standard, the fold change (0.01) represents the FDR between two samples by the use of a corrected *P*-value denoting significant differences. The gene functions were annotated on the basis of the content from six databases: the Nr, Pfam, KOG/COG, Swiss-Prot, KEGG, and GO databases.

### Phylogenetic tree construction

Predicted proteins from the genome were scanned using HMMER v3 (http://hmmer.org/download.html) employing the hidden Markov model (HMM) corresponding to the Pfam GH family. From the proteins obtained using the raw HMM, a high-quality protein set (*E*-value < 1 × 10 − 20 and manual verification of an intact domain) was aligned and used to construct a specific GH family HMM using hmmbuild from the HMMER v3 suite. With this new specific HMM, all proteins with an *E*-value lower than 0.01 were selected. The GH genes were further filtered based on manual curation and functional annotations (supplied by Beijing Biomarker).

## Supplementary information

Supplementary

Supplementary Table 3

Supplementary Table 5

## Data Availability

The raw genome and transcriptome sequencing data have been deposited into the NCBI Sequence Read Archive (SRA) database under BioProject accession numbers PRJNA640193 and PRJNA649380.
