# Supplementary material for: The genome and transcriptome analysis of snake gourd provide insights into its evolution and fruit development and ripening
Source: Hortic Res. 2020 Dec 1;7:199. doi: 10.1038/s41438-020-00423-9 (PMC7704671; doi:10.1038/s41438-020-00423-9)
Supplement: Supplementary file 1 — Supplementary [file 41438_2020_423_MOESM1_ESM.docx]

**Supplementary Table 1 Statistics of the eleven snake gourd assembled chromosomes.**

| **Group** | **Cluster number** | **Cluster length (bp)** | **Order number** | **Order length (bp)** |  |
| --- | --- | --- | --- | --- | --- |
| LG01 | 20 | 118,824,556 | 16 | 117,340,921 |  |
| LG02 | 50 | 101,140,878 | 31 | 97,151,229 |  |
| LG03 | 36 | 84,188,768 | 25 | 82,121,553 |  |
| LG04 | 9 | 88,583,139 | 8 | 88,476,915 |  |
| LG05 | 10 | 86,309,246 | 8 | 86,121,725 |  |
| LG06 | 4 | 80,756,682 | 4 | 80,756,682 |  |
| LG07 | 26 | 75,500,133 | 18 | 74,933,665 |  |
| LG08 | 6 | 76,086,781 | 5 | 75,958,654 |  |
| LG09 | 20 | 74,752,454 | 16 | 74,293,044 |  |
| LG10 | 10 | 68,557,366 | 7 | 68,230,685 |  |
| LG11 | 6 | 64,047,999 | 6 | 64,047,999 |  |
| Total (Ratio %) | 197 (97.52) | 918,748,002 (99.89) | 144 (73.1) | 909,433,072 (98.99) |  |

**Supplementary Table 2 Statistics of repetitive sequences in the snake gourd genome.**

| **Type** | **Number** | **Length** | **Rate (%)** |
| --- | --- | --- | --- |
| ClassI | 950,082 | 691,841,654 | 75.22 |
| ClassI/DIRS | 44,213 | 49,653,121 | 5.40 |
| ClassI/LARD | 244,824 | 93,943,733 | 10.21 |
| ClassI/LINE | 17,404 | 6,826,556 | 0.74 |
| ClassI/LTR/Copia | 412,960 | 419,411,108 | 45.60 |
| ClassI/LTR/Gypsy | 183,418 | 181,966,446 | 19.78 |
| ClassI/LTR/Unknown | 33,204 | 12,498,350 | 1.36 |
| ClassI/PLE | 1,583 | 743,868 | 0.08 |
| ClassI/SINE | 6,053 | 1,080,752 | 0.12 |
| ClassI/TRIM | 6,054 | 1,976,354 | 0.21 |
| ClassI/Unknown | 369 | 87,387 | 0.01 |
| ClassII | 69,374 | 31,112,679 | 3.38 |
| ClassII/Crypton | 447 | 100,816 | 0.01 |
| ClassII/Helitron | 918 | 108,051 | 0.01 |
| ClassII/MITE | 8,772 | 2,083,508 | 0.23 |
| ClassII/Maverick | 680 | 542,404 | 0.06 |
| ClassII/TIR | 52,675 | 26,848,000 | 2.92 |
| ClassII/Unknown | 5,882 | 1,749,789 | 0.19 |
| PotentialHostGene | 21,240 | 4,688,544 | 0.51 |
| SSR | 10,219 | 1,645,226 | 0.18 |
| Unknown | 124,269 | 35,475,007 | 3.86 |
| Total | 1,175,184 | 736,142,385 | 80.03 |

**Supplementary Table 4 Statistics of gene family clustering in the snake gourd.**

| **Item** | **Number** | **Percentage (%)** |
| --- | --- | --- |
| Genes | 22874 | -- |
| Genes in orthogroups | 21894 | 95.7 |
| Unassigned genes | 980 | 4.3 |
| Orthogroups | 17057 | 49.0 |
| Species-specific orthogroups | 125 | -- |
| Genes in species-specific orthogroups | 451 | 2.0 |


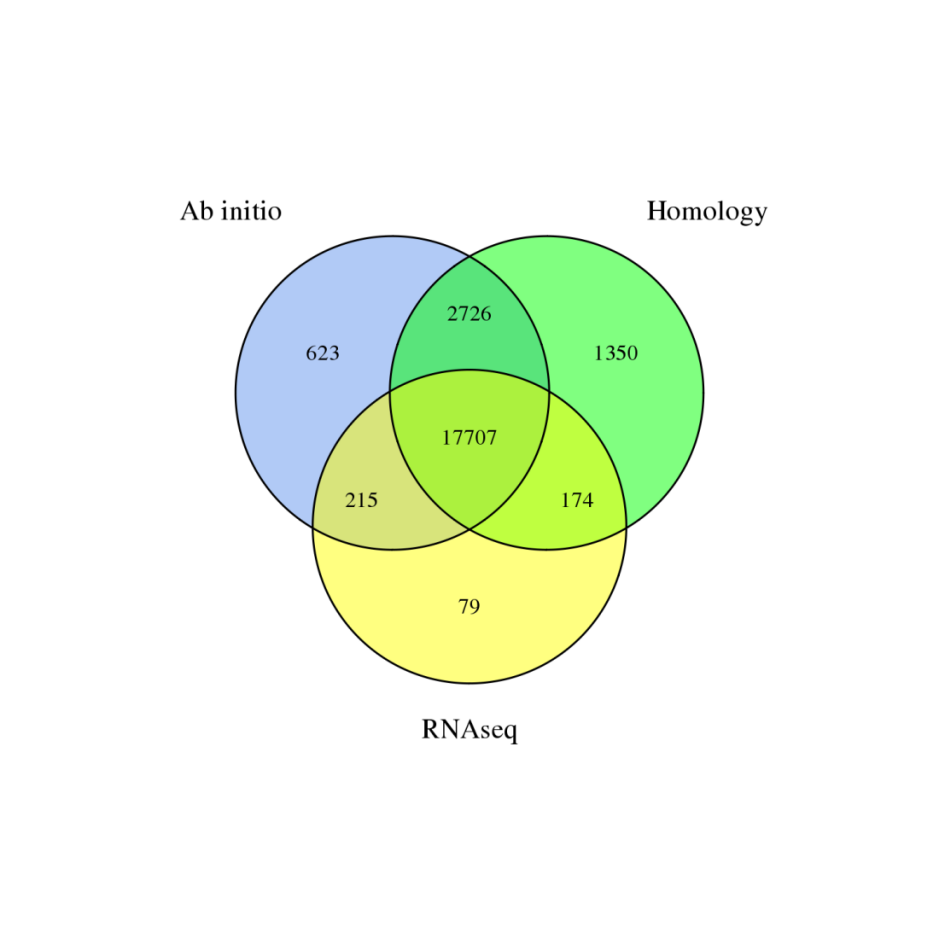


**Supplementary Fig. 1 Distribution of genes derived from three prediction methods after EVM integration.**


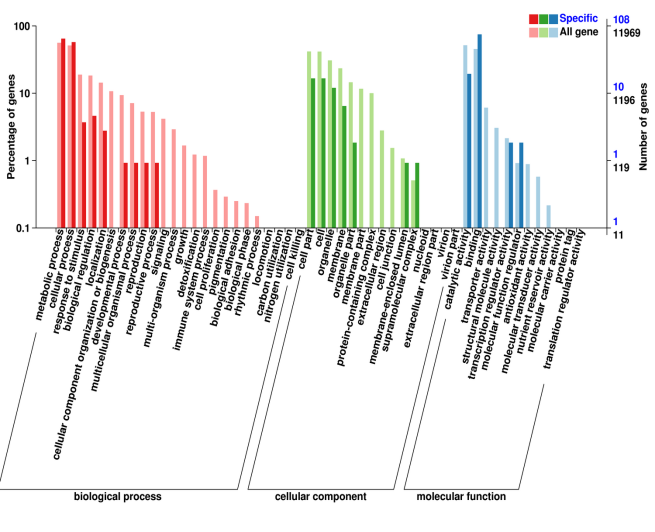

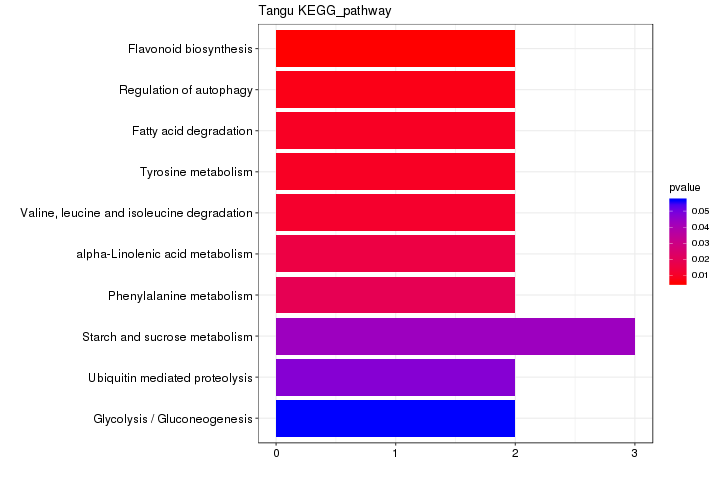


A

B

**Supplementary Fig. 2 (A) KEGG and (B) GO enrichment analyses for gene families specific to snake gourd.**


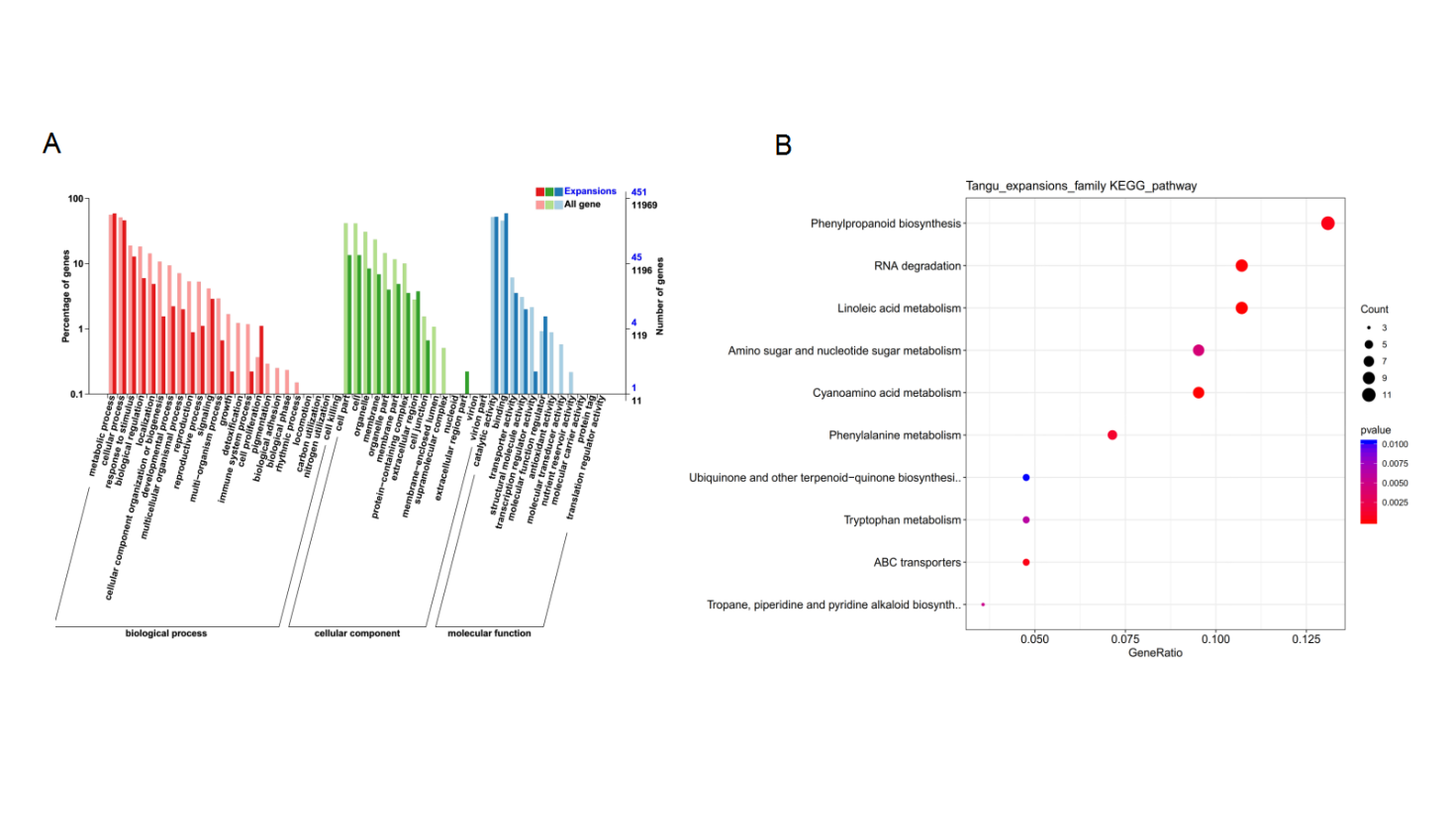


**Supplementary Fig. 3 (A) GO and (B) KEGG enrichment analyses for the expanded genes in the snake gourd genome.**


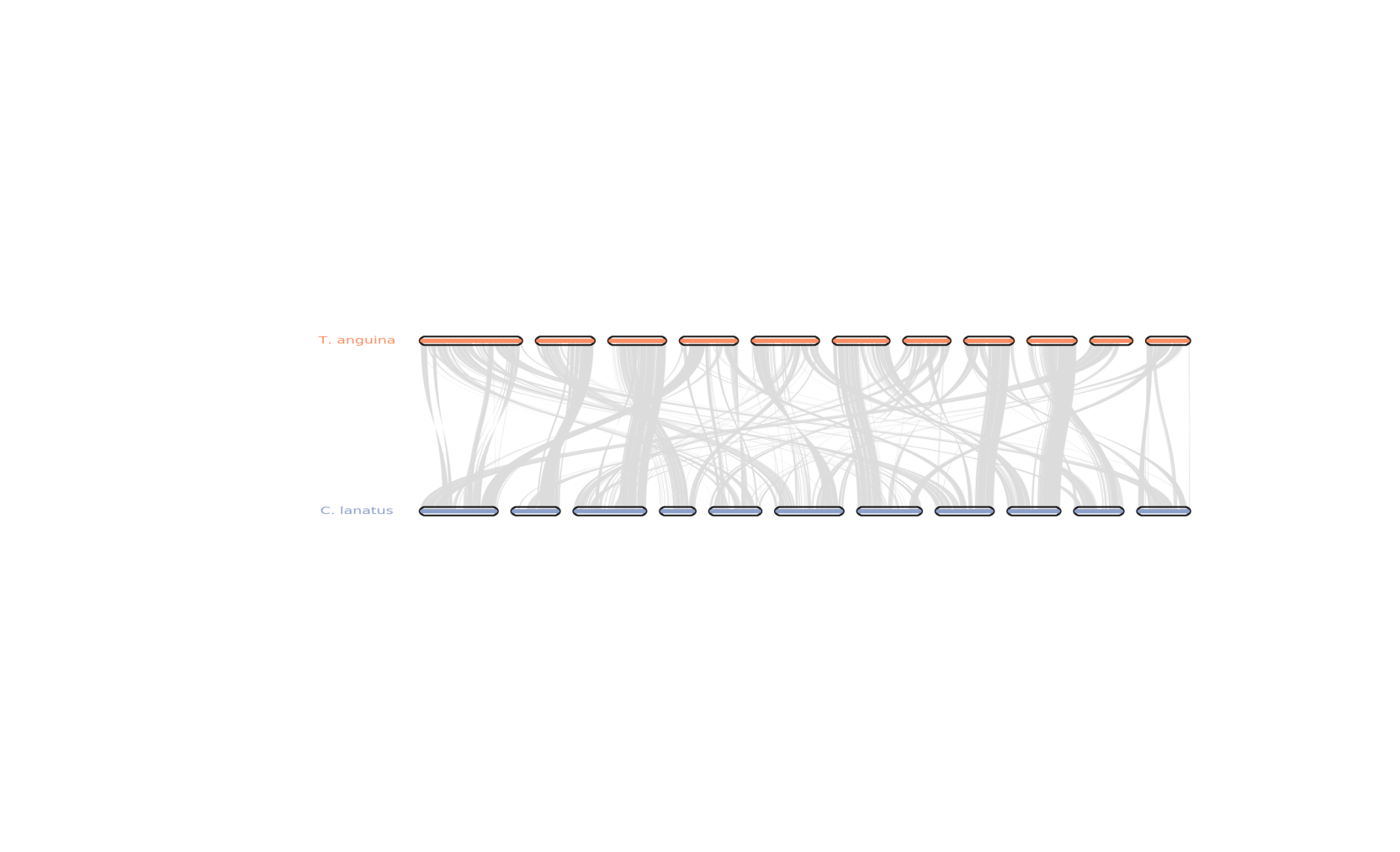

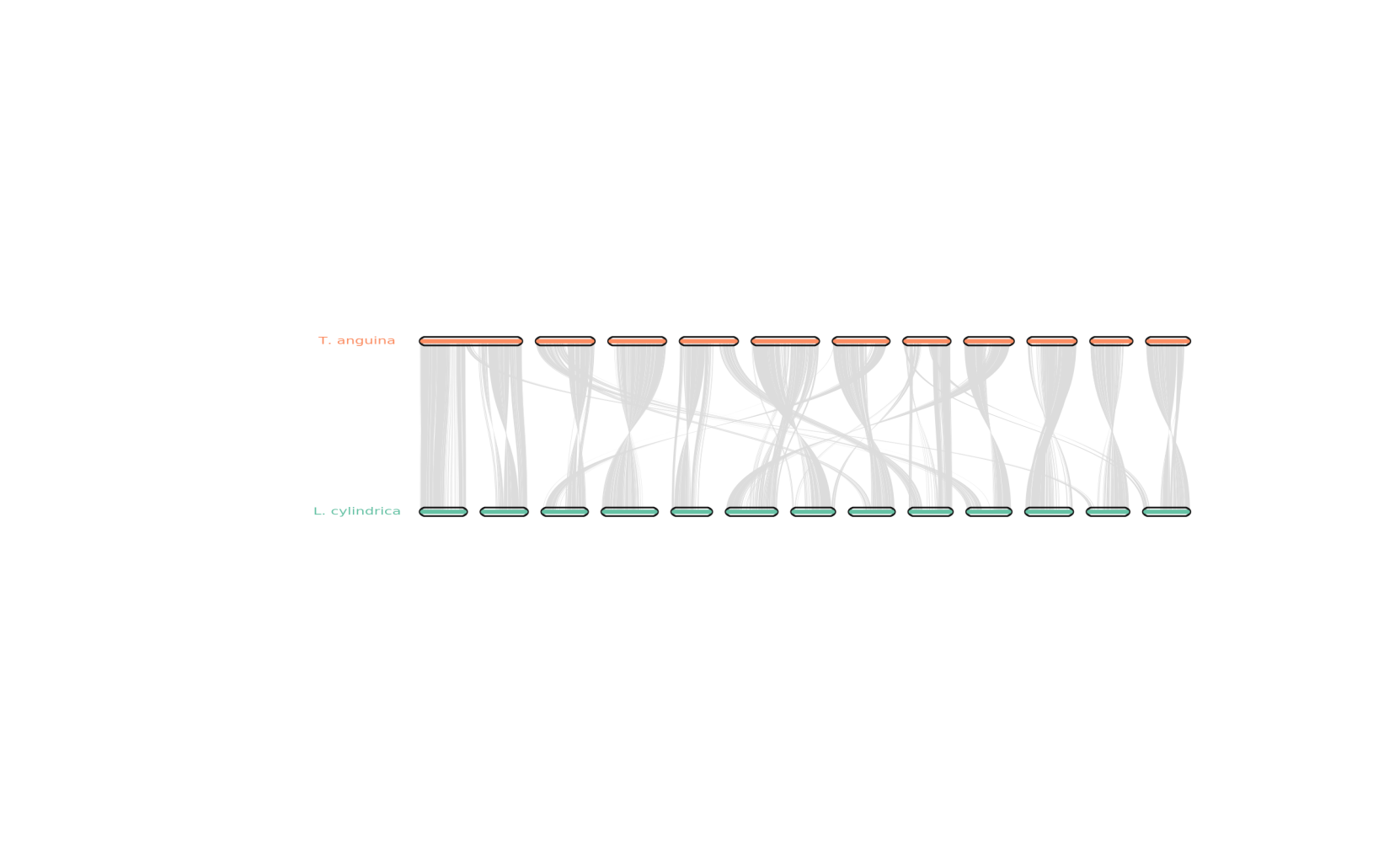


A

B

**Supplementary Fig. 4 Collinearity between genomes of snake gourd and sponge gourd (A), and snake gourd and watermelon (B).**
